# Avoiding Invasive Measures: Sphenopalatine Ganglion Block as a Substitute for Epidural Blood Patch in Post-dural Puncture Headache: A Case Report

**DOI:** 10.5812/aapm-148291

**Published:** 2024-09-08

**Authors:** Saeede Babaiyan, Fatemeh Shakhs Emampour

**Affiliations:** 1 Department of Anesthesiology, School of Medicine, Birjand University of Medical Science, Birjand, Iran; 2 Associate Professor of Anesthesiology,Department of Anesthesiology, School of Medicine Birjand University of Medical Sciences

**Keywords:** Sphenopalatine Ganglion Block, Post-dural Puncture Headache, Spinal Anesthesia, Epidural Blood Patch

## Abstract

**Introduction:**

Post-dural puncture headache (PDPH) is a well-known consequence of neuraxial anesthesia that can impede patient recovery and delay early discharge. Traditional remedies include hydration and the administration of simple analgesics for symptom relief. When symptoms persist despite conservative interventions, an epidural blood patch (EBP) is typically recommended. However, this invasive procedure carries risks and complications. Our case report aims to explore a potential alternative treatment for PDPH.

**Case Presentation:**

We present the case of a 22-year-old female who experienced PDPH following spinal anesthesia. Despite initial attempts at conservative management, her symptoms persisted. She then opted for a trans-nasal sphenopalatine ganglion (SPG) block, which resulted in remarkable pain relief and eliminated the need for an EBP.

**Conclusions:**

The SPG block emerges as a minimally invasive option for treating PDPH. Multiple studies have demonstrated that patients undergoing SPG block therapy did not require EBP.

## 1. Introduction

Over one-third of women experience headaches within a week after childbirth, with 75% being benign, such as migraines. The remainder are secondary, resulting from vascular issues, hypertension, infections, or dural puncture (1). Post-dural puncture headache (PDPH) arises as a complication from either unintentional or deliberate dural puncture during procedures such as epidural analgesia, spinal anesthesia, or other neuraxial interventions (2). Its incidence fluctuates between 2% and 40%, influenced by various procedural and patient-related factors (3). Post-dural puncture headache typically presents as a headache exacerbated by changes in posture, often accompanied by neck stiffness and pain (2). The severity of PDPH can disrupt daily activities and baby care, especially in postpartum patients (4).

**Figure 1.**
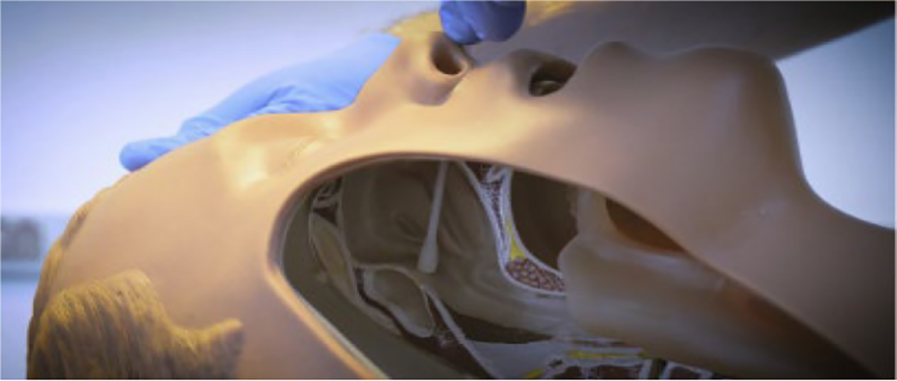
Trans-Nasal approach for sphenopalatine ganglion block procedure (11)

Initial management involves analgesics, hydration, and avoiding changes in position. Oral or intravenous caffeine may be considered, though its efficacy is debatable. Experimental treatments like steroids, ACTH, and gabapentin have shown inconclusive results (5). The epidural blood patch (EBP), introduced in 1960, remains the gold standard with success rates ranging from 75% to 90% (6, 7). The sphenopalatine ganglion (SPG) has been used in the treatment of conditions like migraines and cluster headaches. The SPG block shows potential in managing PDPH by blocking parasympathetic flow to the cerebral vasculature, which may relieve the headache (8-10). This report discusses our successful application of the SPG block in managing a patient. Figure 1 illustrates the SPG block (11).

## 2. Case Presentation

Our patient was a 22-year-old female, weighing 55 kg, who was referred for a cesarean section at 37 weeks of pregnancy due to breech presentation of the fetus. After receiving 500 cc of isotonic normal saline, the patient underwent spinal anesthesia while in a sitting position.

### 2.1. Preparation

Spinal anesthesia was performed using a Quincke type 25 G spinal needle, successfully on the first attempt, with clear, free-flowing cerebrospinal fluid. A combination of 2.5 mg bupivacaine 5% and 25 μg fentanyl was administered, resulting in effective analgesia. Vital signs remained normal during the operation and in the ward. The patient did not report a headache during the initial hospitalization and was discharged the following day in good general condition. However, three days after the operation, the patient experienced an unbearable headache and neck pain, which worsened with sitting and standing but was relieved by lying down. She described the headache as localized and severe, rating it 9/10 on the numerical rating scale (NRS) (12). The patient did not report other neurological symptoms, blurred vision, nausea, or vomiting. Examination of the cranial nerves was normal. Following a neurologist's consultation, a brain CT scan was performed to rule out other causes of the headache or subdural bleeding related to intracranial hypotension. The brain CT scan showed normal results.

### 2.2. Treatment

Intravenous hydration was initiated, and the patient was prescribed simple pain relievers (ketorolac 30 mg and diclofenac suppositories). Within 1 hour, the headache decreased from NRS 9/10 to 8/10.

**Figure 2.**
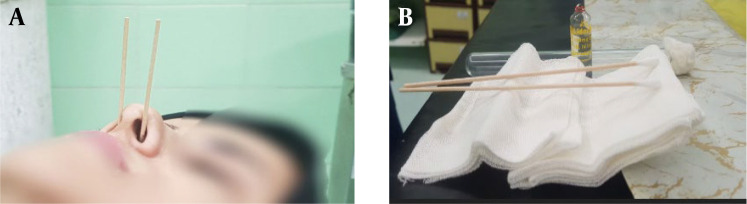
Sphenopalatine ganglion block procedure

Based on the initial diagnosis of PDPH and with the patient's consent, an SPG block was performed using an intranasal approach. This procedure involved saturating a cotton-tip applicator with 2% lidocaine and inserting it into the posterior nasopharynx. After 10 minutes, the cotton tip was removed, re-soaked with lidocaine, and repositioned for an additional 10 minutes (Figure 2). 

This resulted in a further reduction in headache intensity from NRS 8/10 to 3/10 within 5 minutes. Twelve hours after the procedure, her neck pain and headache had decreased, and she was able to perform her activities independently. After 24 hours, the patient was discharged with a tolerable headache. In a telephone follow-up 48 hours after discharge, the patient reported significant improvement in the headache, with an NRS score of 2/10.

## 3. Discussion

Post-dural puncture headache usually appears within 5 days of a dural puncture (2, 13). Its cardinal features include a postural nature, which improves when lying down and worsens when sitting or standing. It is often accompanied by neck stiffness and pain, photophobia, tinnitus, nausea, and vomiting. Various risk factors contribute to PDPH, such as young age, female sex, pregnancy, and characteristics of the needle used, including type, size, and orientation. Cutting needles (Quincke) are associated with a higher incidence of PDPH compared to blunt or pencil-point needles (Sprotte, Whitacre), and larger bore needles also increase the risk. Proper needle orientation parallel to the spine's long axis minimizes dural disruption, thereby reducing the risk of PDPH (14).

The mechanism of PDPH remains unclear, but one theory suggests that continuous cerebrospinal fluid (CSF) leakage through the dural tear results in reduced intracranial volume. Compensatory vasodilation, mediated by parasympathetic activity, attempts to restore intracranial volume, resulting in a throbbing headache. Even after vasodilation, parasympathetic activity persists. The SPG, located within the pterygopalatine fossa, is an extracranial parasympathetic ganglion. Blocking the SPG inhibits parasympathetic activity and reduces vasodilation. The SPG block has shown efficacy in headache treatment (15).

The trans-nasal SPG block is a safe and straightforward procedure. It involves inserting a soaked applicator with a local anesthetic (such as 2% lidocaine) parallel to the nasal floor until resistance is met, reaching the posterior pharyngeal wall. Although direct contact with the ganglion is not achieved, the local anesthetic permeates around it, facilitated by connective tissue and mucous membranes. This block does not address CSF leaks, so supportive measures, including bed rest, analgesics, hydration, and laxatives, are necessary until pain relief is complete. Kent and Mehaffey used SPG blocks in three patients after spinal analgesia. All patients experienced prompt headache resolution, though two of the three had recurrent headaches and required repeat SPG blocks (10). Cohen et al. studied a larger cohort and reported significant improvement in headaches at 30 and 60 minutes after SPG block compared to EBP (39% vs. 21% and 71% vs. 31%, respectively). No significant difference was observed in headache reduction at 24 hours, 48 hours, or 1-week post-treatment between the two groups. Additionally, no significant complications were noted in the SPG block group, whereas patients treated with EBP experienced back pain (7.7%), hypotension (2.6%), and temporary hearing disorders (2.6%). This study suggests that SPG block is a safe alternative for PDPH treatment (10, 16). Asmara et al. described successful PDPH treatment with SPG block in a post-cesarean patient, a 26-year-old woman (17).

In summary, while EBP is considered the gold standard for managing PDPH, its invasive nature poses potential risks, including unintended dural puncture and complications such as meningitis, arachnoiditis, seizures, hearing disorders, and vision problems. In contrast, the SPG block offers a promising, minimally invasive alternative. The ability to repeat the SPG block as needed and the potential to avoid EBP provides significant advantages, minimizing risks and enhancing patient satisfaction. The SPG block, alongside general supportive measures, can be considered early in PDPH treatment. However, if pain persists despite these measures, EBP remains a viable option.

### 3.1. Conclusions

We recommend that individuals experiencing PDPH consider undergoing the less invasive SPG block procedure, which has minimal adverse effects. In many cases, this method may eliminate the need for a blood patch and its associated side effects and complications.

## Data Availability

The data is available on request from corresponding author.
